# Supplementary material for: Acceptance and compliance with micronutrient powder (MNP) among children aged 6–23 months in northern Nigeria
Source: PLOS Glob Public Health. 2022 Oct 17;2(10):e0000961. doi: 10.1371/journal.pgph.0000961 (PMC10022258; doi:10.1371/journal.pgph.0000961)
Supplement: S1 Dataset — (ZIP) [file pgph.0000961.s008.zip › S8_Dataset/Direct Observations.zip!/Direct Observations/280615_A_GU_ID_1_DO.pdf]

1.1.1 Instrument F –Direct Observation Form

**<<Instrument F Direct Observation Form>>**
**PI Name:** PragyaMathema,Stephen Kodish<br>**Study Title:** Formative research to develop an infant and young child feeding (IYCF) intervention with micronutrient powder (MNP) for children aged 6 – 23 months<br>**PI Version/Date:** Version1.0/January 24, 2015

Date: ___28TH JUNE, 2015_________________Location: _____GUGU, GUYUK L.G.A_____________

Data Collector: ___ANTIYA SANDA______________Participant ID: _A HOUSE NEAR A GRINDING MACHINE

Main goals of this direct observation are to answer the following questions:
1. In this household, what would be the best way(s) to give nutritional supplements to young children? Why?
2. In this household, describe the food sharing that you observe throughout the meal. Are there any predictable patterns?
3. In this household, who is favored and how is this favoritism expressed during meal preparation and eating?

(TEAR OFF AT PERFORATED DOTTED LINE BELOW)

PARTICIPANT ID: __________

Household Composition Table (Fill out as you do data collection)

INITIALS	SEX (M/F)	AGE (estimated)	Relationship to household	Description
FB	M	40 YEARS	HUSBAND	A FAT BLACK MAN IN HIS MIDDLE 40'S
TF	F	33 YEARS	WIFE	A TALL FAIR WOMAN WEARING AN ORANGE WRAPPER
CB	F	6 MONTHS	DAUGHTER	A CUTE LITTLE BABY
SL	M	25 YEARS	RELATIVE	A SLIM YOUNG BOY WEARING A LONG TROUSER
SW	F	30 YEARS	RELATIVE	A SLIM WOMAN WEARING A PINK BLOUSE.

PARTICIPANT ID: __________

TIME	ACTOR INITIALS	RECIPIENT INITIALS	WHAT HAPPENED	FOOD	Reactivity
8:00	TF		IS SWEEPING THE HOUSE		
8:05	TF		IS SWEEPING THE HOUSE		
8:10	FB, CB		IS CARRYING THE BABY ON HIS ARM		
8:15	TF		GATHERS PLATES TO WASH		
8:20	TF		IS WASHING THE DIRTY PLATES		
8:25	TF		STARTS MAKING FIRE		
8:30	TF		CARRYS A POT AND POURS WATER INTO IT	WATER	
8:35	TF		PUTS THE POT WITH WATERINSIDE ON THE FIRE	WATER	
8:40	TF		COVERS THE POT		
8:45	TF		BRINGS A SMALL BUCKET		
8:50	TF		POURS HOT WATER INTO THE		

TIME	ACTOR INITIALS	RECIPIENT INITIALS	WHAT HAPPENED	FOOD	Reactivity
			BUCKET		
8:55	TF		MIXES HOT WATER AND COLD WATER IN A BUCKET	WATER	
9:00	TF		BRING A HOT WATER FLASK		
9:05	TF		POURS HOT WATER INTO THE FLASK		
9:10	TF		CARRYS THE BUCKET OF HOT AND COLD WATER		
9:15	TF, CB		COLLECT THE BABY FROM THE FATHER		
9:20	TF	CB	IS REMOVING HER BABY'S CLOTHE		
9:25	TF	CB	STARTS BATHING THE BABY		
9:30	TF	CB	IS BATHING THE BABY WITH BATHING SOAP AND SPONGE		
9:35	TF	CB	IS CLEANING THE BABY'S BODY WITH A TOWEL		
9:40	TF	CB	STARTS APPLYING CREAM ON THE BODY OF THE BABY		
9:45	TF		GIVES THE BABY TO THE FATHER		
9:55	TF		BRINGS THE HOT WATER FLASK AND POUR WATER INTO THE POT		
10:00	TF		BRINGS GUINEA CORN FLOUR, SOYA BEANS FLOUR AND GROUNDNUT PASTE	GUINEA CORN, SOYA BEAN AND GROUNDNUT	
10:01	TF		STARTS MIXING THE GUINEA CORN FLOUR, SOYA BEANS FLOUR AND GROUNDNUT PASTE TOGETHER IN A BOWL	GUINEA CORN, SOYA BEANS AND GROUNDNUT	
10:02	TF		POURS WATER INTO THE POT ON THE FIRE	GUINEA CORN, SOYA BEANS AND GROUNDNUT	
10:03	TF		STARTS STIRRING THE MIXTURE ON THE FIRE	GUINEA CORN, SOYA BEANS AND GROUNDNUT	

TIME	ACTOR INITIALS	RECIPIENT INITIALS	WHAT HAPPENED	FOOD	Reactivity
10:04	TF		CONTINUES TO STIR THE GUINEA CORN FLOUR, SOYA BEANS FLOUR AND THE GROUNDNUT PASTE TOGETHE	GUINEA CORN, SOYA BEANS AND GROUNDNUT	
10:05	TF		BRINGS A SMALL CUP		
10:06	TF		FETCHES THE KUNU INTO A SMALL CUP	GUINEA CORN	
10:07	TF		KEEPS THE SMALL CUP ON THE GROUND		
10:08	TF		BRINGS THE POT DOWN FROM THE FIRE		
10:09	TF		PICKS THE SMALL CUPP WITH KUNU INSIDE AND BEGINS TO STIR USING A SPOON		
10:10	TF		GOES INTO THE ROOM TO BRING THE MNP		
10:15	TF		ALLOWS THE KUNU TO GET COLD BEFORE UNSEALING THE MNP		
10:20	TF		TASTE THE KUNU TO KNOW IF IT IS COLD BEFORE POURING THE MNP		
10:25	TF		POURS MNP INTO THE KUNU	MNP AND GUINEA CORN	
10:30	TF		STARTS MIXING THE MNP INTO THE KUNU		
10:35	TF		LOOKS INTO THE CUP AND DISCOVER THAT THE MNP HAS BLENDED WELL WITH THE KUNU	GUINEA CORN AND MNP	
10:40	TF		COLLECTS THE BABY FROM THE FATHER		
10:45	TF	CB	STARTS FEEDING THE BABY WITH THE KUNU MIXED WITH THE MNP	GUINEA CORN AND MNP	
10:50	TF	CB	IS FEEDING THE BABY WITH THE KUNU	GUINEA CORN AND MNP	
10:55	TF	CB	IS FEEDING THE BABY WITH THE KUNU	GUINEA CORN AND	

TIME	ACTOR INITIALS	RECIPIENT INITIALS	WHAT HAPPENED	FOOD	Reactivity
				MNP	
11:00	TF	CB	GIVES THE BABY THE LAST SPOON	GUINEA CORN AND MNP	
11:05	TF		DROPS THE EMPTY CUP OF KUNUON THE GROUND		
1110	CB		DRANK ALL OF HER KUNU	GUINEA CORN AND MNP	
11:15	TF	CB	CARRYS THE BABY AND GIVES TO THE FATHER		
11:20	TF		FETCHES WATER IN A BUCKET		
11:25	TF		GOES TO THE BATHROOM		
11:30	FB	CB	THE DAD IS WEARING THE BABY CLOTHES		
11:35	FB	CB	THE DAD IS WEARING THE BABY CLOTHES AND IS PLAYING WITH THE CHILD		
11:40	TF		COMES OUT OF THE ROOM WELL DRESSED		
11:45	TF		BIDS THE DAD AND THE DAUGHTER FAREWLL AND LEAVES FOR CHURCH		
11:50	FB	CB	CARRYS THE BABY INSIDE THE ROOMAND KEPT HER ON THE MAT		
11:55	CB		STARTS PLAYING WITH HER TOYS		
12:00	CB		IS PLAYING ON THE MAT		
12:05	CB		PLAYING		
12:10	CB		PLAYING		
12:15	CB		PLAYING		
12:20	CB		STARTS CRYING		
12:25	FB	CB	CARRYS THE BABY ON THE ARM		

TIME	ACTOR INITIALS	RECIPIENT INITIALS	WHAT HAPPENED	FOOD	Reactivity
12:30	FB		BRINGS A CUP AND SPOON		
12:31	FB		BRINGS THE HOT WATER FLASK	WATER	
12:32	FB		POURS HOT WATER INTO A BABY'S FEEDING BOTTLE	WATER	
12:33	FB		BRINGS A CONTAINER OF POWDERED MILK (NAN)	MILK	
12:34	FB		FETCHES 3 SPOONS OF NAN AND POURS INTO THE BABY'S FEEDING BOTTLE		
12:35	FB		BEGINS TO SHAKE THE BABY'S FEEDING BOTTLE	MILK	
12:40	FB	CB	CARRY'S BABY ON THE LAP		
12:45	FB	CB	STARTS FEEDING THE BABY WITH THE POWDERED MILK MIXED WITH HOT WATER	MILK	
12:50	FB	CB	CONTINUES TO FEED THE BABY USING AN INFANT FEEDING BOTTLE	MILK	
12:55	FB	CB	IS FEEDING THE BABY	MILK	
1:00	CB		STARTS SLEEPINGON THE FATHERS LAP		
1:05	FB	CB	CARRYS THE BABY ON THE ARMS		
1:10	FB	CB	LAYS THE BABY ON THE MAT		
1;10	CB		IS SLEEPING		
1;15	CB		IS SLEEPING		
1:20	CB		IS SLEEPING		
1:25	CB		IS SLEEPING		
1:30	CB		IS SLEEPING		
1:35	CB		SLEEPING		

TIME	ACTOR INITIALS	RECIPIENT INITIALS	WHAT HAPPENED	FOOD	Reactivity
1:40	CB		SLEEPING		
1:45	CB		SLEEPING		
1:50	CB		SLEEPING		
1:55	CB		SLEEPING		
2:00	CB		SLEEPING		
2:05	CB		SLEEPING		
2:10	TF		WALKS INTO THE HOUSE		
2:15	TF		GOES INTO THE ROOM		
2:20	TF		COMES OUT OF THE ROOM		
2:25	TF	CB	COVERS HER BABY WITH A WRAPPER		
2:30	TF		STARTS MAKING FIRE		
2:31	TF		PUTS A POT ON THE FIRE		
2:32	TF		POURS PALM OIL INTO THE EMPTY POT ON THE FIRE	PALM OIL	
2:33	TF		PICKS AN ONION AND STARTS SLICING IT	ONION	
2:34	TF		POURS ONIONS INTO THE HOT PALM OIL	PALM OIL	
2:35	TF		USES A SPOON TO STIR THE ONIONS AND PALM OIL	PALM OIL AND ONIONS	
2:40	TF		BRINGS SOME TINY DRY FISH	FISH	
2:41	TF		CHOPS THE DRY FISH INTO THE POT ANDPOURS WATER	FISH AND WATER	
2:42	TF		ADDS SEASONING INTO THE POT	MAGGI	
2:43	TF		ADDS SALT INTO THE POT	SALT	
2:44	TF		COVERS THE POT		

TIME	ACTOR INITIALS	RECIPIENT INITIALS	WHAT HAPPENED	FOOD	Reactivity
2:45	TF		BRINGS A BIG BOWL OF MAIZE FLOUR	MAIZE	
2:50	TF		STARTS SIEVING MAIZE FLOUR	MAIZE	
2:55	TF		CONTINUES TO SIEVE THE MAIZE FLOUR	MAIZE	
2:56	SW		WALKS INTO THE HOUSE AND HELPS THE WIFE TO SIEVE		
2:57	SW		IS SIEVING THE MAIZE FLOUR	MAIZE	
2:58	TF		OPENS THE POT ON THE FIRE AND TAST THE FISH TO KNOW IF IT IS SOFT	FISH	
2:59	TF		STARTS POURING DRY OKRO INTO THE POT ON THE FIRE	OKRO	
3:00	TF		IS POURING DRY OKRO AND STIRRING THE SOUP	OKRO	
3:01	TF		IS STIRRING THE OKRO SOUP	OKRO	
3:02	TF		BRINGS DOWN THE SOUP POT FROM THE FIRE	OKRO	
3:03	TF		COVERS THE POT		
3:04	TF		POURS WATER INTO ANOTHER EMPTY POT	WATER	
3:05	TF		PUTS THE EMPTY POT WITH WATER ON THE FIRE	WATER	
3:06	TF		COVERS THE POT ON THE FIRE		
3:07	TF		FETCHES THE SIEVED MAIZE FLOUR IN A BOWL AND POURS WATER ON IT	MAIZE AND WATER	
3:08	TF		POURS MIXED MAIZE WITH WATER INTO THE HOT WATER ON THE FIRE	MAIZE AND WATER	
3:09	TF		USES A STICK TO MIX THE MAIZE ON THE FIRE		
3:10	TF		REMOVES THE STICK AND COVERS THE POT		
3:11	TF		GOES INTO THE KITCHEN TO BRING FOOD WARMERS AND A SERVING PLATE		
3:12	TF		OPENS THE POT ON THE FIRE AND POURS MAIZE FLOUR INTO		

TIME	ACTOR INITIALS	RECIPIENT INITIALS	WHAT HAPPENED	FOOD	Reactivity
			IT		
3:13	TF		STARTS MIXING THE MAIZE FLOUR FASTER		
3:14	TF		CONTINUES TO MIX THE MAIZE FLOUR FAST		
3:15	TF		IS MIXING THE MAIZE INTO TUWO		
3:16	TF		REMOVES THE TUWO STICK		
3;17	TF		STARTS SERVING TUWO INTO THE FOOD WARMER		
3:18	TF		IS SERVING THE TUWO INTO A PLATE		
3:19	TF		COVERS THE FOOD WARMER		
3:20	TF		BRINGS THE POT DOWN FROM THE FIRE		
3:21	TF		POURS WATER INTO THE EMPTY POT		
3:22	TF		OPENS THE POT OF SOUP	OKRO	
3:23	TF		SERVES THE SOUP INTO A SMALL SOUP BOWL	OKRO	
3:24	TF		TAKES THE SOUP BOWL AND THE FOOD WARMER TOGETHER INTO THE ROOM		
3:25	TF		SERVES HER HUSBAND THE FOOD AND COLECTS THE BABY FROM HIM	OKRO AND MAIZE	
3:30	TF		COMES OUT OF THE ROOM CARRYING HER BABY		
3:35	TF	SW	CARRY'S A PLATE OF TUWO AND GIVES HER RELATIVE	OKRO AND MAIZE	
3:40	TF		GOES INTO THE ROOM		
3:45	TF, FB		STARTS EATING TOGETHER	OKRO AND MAIZE	
3:50	TF, FB		ARE EATING TUWO TOGETHER	OKRO AND MAIZE	
3:55	TF, FB		ARE EATING TOGETHER AND CHATTING	OKRO AND MAIZE	
4:00	TF, FB		ARE EATING TOGETHER	OKRO AND MAIZE	
4:05	TF, FB		ARE EATING TOGETHER	OKRO AND MAIZE	

TIME	ACTOR INITIALS	RECIPIENT INITIALS	WHAT HAPPENED	FOOD	Reactivity
4:10	TF, FB		ARE EATING TOGETHER	OKRO AND MAIZE	
4:15	TF, FB		ARE EATING TOGETHER	OKRO AND MAIZE	
4:20	TF, FB		ARE EATING TOGETHER	OKRO AND MAIZE	
4:25	TF, FB		ARE EATING TOGETHER	OKRO AND MAIZE	
4:30	TF		STARTS PACKING THE EMPTY PLATES		
4:35	TF		TAKES EMPTY PLATES OUTSIDE		
4:40	TF		KEEPS EMPTY PLATES IN THE KITCHEN		
4:45	TF, SW		STARTS CHATTING		
4:50	TF, SW		CHATTING TOGETHER		
4:55	TF, SW		CHATTING TOGETHER		
5:00	TF, SW		CHATTING TOGETHER		
5:10	TF, SW		CHATTING TOGETHER		
5:15	TF		BRINGS A HOT WATER FLASK	WATER	
5:20	TF		POURS HOT WATER INTO THE BABY'S FEEDING BOTTLE AND ADD POWDERED MILK AND SHAKES IT	MILK	
5:25	TF	CB	CARRYS HER BABY ON THE LAP		
5:30	TF	CB	STARTS FEEDING HER CHILD WITH MILK		
5:35	TF	CB	IS FEEDING THE BABY		
5:40	CB		STARTS SLEEPING		
5:45	TF	CB	CARRYS HER CHILD TO THE ROOM		
5:50	CB		IS SLEEPING		
5:55	CB		IS SLEEPING		
6:00	CB		IS SLEEPING		
6:05	TF		COMES OUT OF THE ROOM AND CONTINUES TO CHAT WITH HER RELATIVE		

TIME	ACTOR INITIALS	RECIPIENT INITIALS	WHAT HAPPENED	FOOD	Reactivity

PARTICIPANT ID: _________

Primary Questions (Complete after you complete data collection):

From what you have seen observing THIS HOUSEHOLD, please answer the following questions in detail:

Question	Write your detailed response here
1. In this household, what would be the best way(s) to give nutritional supplements such as an MNP to young children to ensure compliance?<br><br>Why?	The best way to give the MNP to the young child in this household would be in the morning. That is when the child will be hungry and would comply easily.<br><br>The young child here wakes up very hungry and does not rejects the kunu given to her, because when she is filled with her milk, she wouldn't agree to eat any other complimentary food.
2. In this household, who is	The husband as the head of the house is favoure during the meal

favored during meal preparation or feeding?<br><br>How is this favoritism expressed?	preparation and the wife also favours herself.<br><br>The husband is served the large share together with the wife because they eat together on the same plate. So the wife serves the larger share to herself and the husband before serving other relatives.
3. How could we promote the MNP supplement for this family that would attract their attention?<br><br>Who would they listen to?	The MNP should be promoted through the father and the mother also because both of them participate in the feeding and upbringing of their baby.<br><br>The baby will listen to both the father and the mother because she is just a little baby and she is already used to the dad and mum. Whatever the dad or mum gives to her, she easily accepts it without rejecting or crying.
4. In this household, what would be the best ways to promote the MNP but limit how much it would be shared?<br><br>Why?	In this household, the best way to promote the MNP would be through the dad because he is aware of everything that happens in the house. MNP could not likely to be shared in this household because the parents are educated and know the instruction written on the MNP pamphlet.<br><br>The parents of this young child are educated and already know how the MNP should be used and the instruction that it should not be shared among neighbours or other children.
5. What other issues and concerns did you see with this family that are important for us to consider with regard to introduction of an MNP?<br><br>Why?	There are no concern relating to hygiene or lack of food or feeding pattern that is of any importance.<br><br>From what I saw today while observing this household, there are no concern to consider with regards to introduction of the MNP.

6. Based on your experience observing this child today, do you believe reactivity negatively impacted your ability to see the food-related behaviors of the child?<br><br>Please explain your answer in detail.	The dad was showing much care to the child in my presence but in general, reactivity did not negatively impact my ability to see the food related behaviours of the child.<br><br>My observation was not in any way affected by the reactivity because reactivity only occurred once.
